# Non motorized trip pattern of high density neigbourhood: Data on demography and socio-economic parameters

**DOI:** 10.1016/j.dib.2018.08.082

**Published:** 2018-08-30

**Authors:** Busari Ayobami, Ojo Oladipupo

**Affiliations:** Department of Civil Engineering, Covenant University, Ogun State, Nigeria

**Keywords:** Sustainable transportation, Travel behavior, Walk trip, Non-motorized trip, Trip pattern

## Abstract

This article analysed data on the effect of demography and socioeconomic parameters on non-motorized trip with special focus on walking as a modal choice. To achieve this aim, 500 detailed question forms were administered to respondents who are 18 years and above in Ota, Ogun State Nigeria. Information on volume of trips, types of trips, modal split, and land use were analyzed. Descriptive and bivariate analysis were done to show the relationship between the parameters using SPSS version 23. The data will be useful for transportation planners, highway engineers, transportation research institute and policy makers on the factors affecting the use of walk in the study area and similar cities in the world.

**Specification table**

**Table:** 

Subject Area	Highway Engineering, Transportation Management
More Specific Subject Area	Travel Behaviour, Trip Pattern
How was data acquired	Questionnaire Analysis, Focus Group
Type of data	Tables and Figures
Data Format	Analysed, Descriptive and Statistical Data
Experimental Factors:	Sample consist of trip pattern of respondents in Ota, Ogun State Nigeria. Effect of demography and socio economic parameters on non-motorized trips are assessed to enhance effective mobility in developing countries.
Experimental Features	The germane factors affecting the choice of walking as a modal choice with special focus on demography, socio-economic parameters and land use were assessed towards sustainable transportation
Data Source Location	Ota, Ogun State, Nigeria
Data Accessibility	The data is available within this article

**Value of data**

• The data presented the effect of demography and socio-economic parameters on walk trip which can be used by transportation planners, policy makers and other researchers.
• The data revealed the effect of socio-economic parameters and land use on walk trip useful for transportation planners and policy makers.
• The data set showed the trip pattern of pedestrian the factors affecting walking as a modal choice which will guide researchers.

## 1 Data

**Fig. 1:**
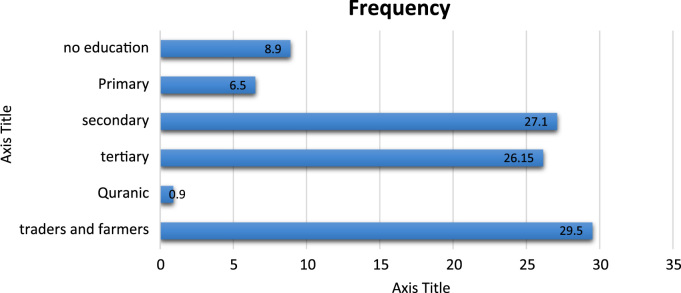
Relationship between walk trip and educational status.

Fig. 1 showed the use of walking as a mode for both work and recreational trip. The result revealed that traders and farmers had the highest percentage of walk trip (Fig. 1).

### 1.1 Effect of land use on walk trip

**Fig. 2:**
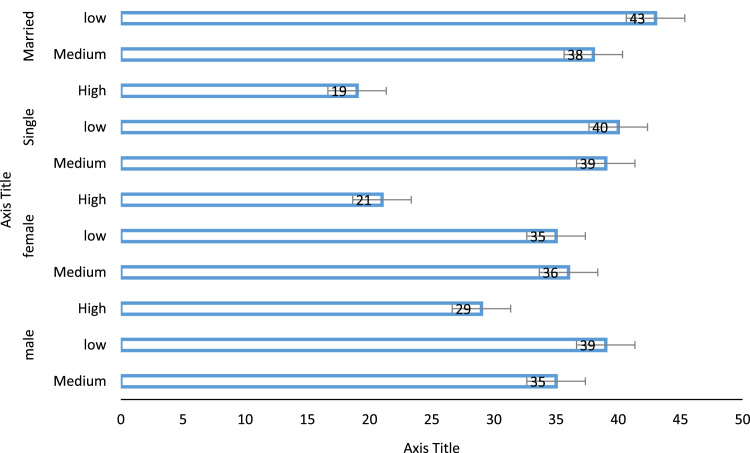
Effect of gender and marital status on work trip.

**Fig. 3:**
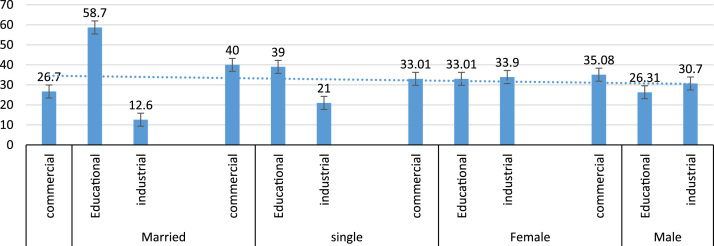
Assessment of trip pattern based on the land use.

**Fig. 4:**
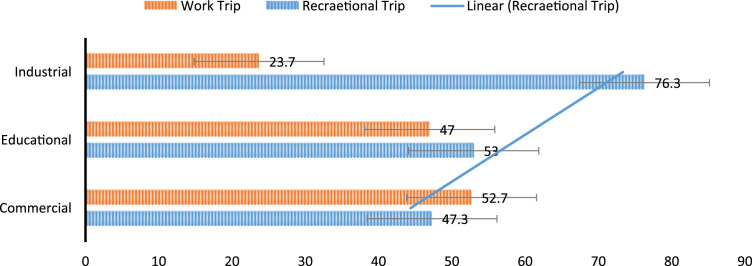
Relationship between land use and trip purpose.

Based on the respondents land use location 58.7% of the married engage in walking for both work and recreational trip in educational zone (Fig. 2). Based on the trip purpose, 76.3% of the respondents in industrial zone uses this mode for recreational trip while 23.7% uses the mode for work trip (Fig. 3 and Fig. 4). Data on other land use and walk trip purpose is also shown.

### 1.2 Assessment of walk trip based on access mode using gender and marital status

**Fig. 5:**
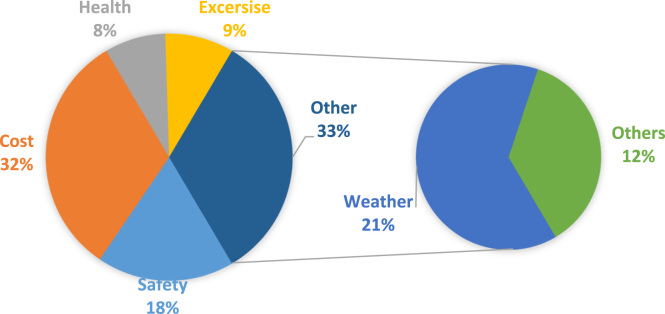
Factors affecting walk trip.

The result of this analysis showed that generally the low income earners embark on more walk trip than other income class (Table 1). The spatial assessment of walk trip based on access mode is presented in (Table 2). The factors affecting the choice of this mode is as shown in Fig. 5.

**Table 1:** Data on walk trip indicating the Access Mode.

	**Income Level**	**Access Mode**	**Access Sub-Mode**	**Circulation/Exchange**
	High	24	20	56
**Male**	Medium	50	38.46	12
	low	30	32	28
	High	45	12	43
**Female**	Medium	36	29	35
	low	43	26	31
	High	30	36	34
**Single**	Medium	31	30.7	38
	low	29	30	41
	High	41	30	29
**Married**	Medium	36	32	32
	low	46	40	14

**Table 2:** Data set on the spatial assessment of walk trip using access mode.

		**Spatial Assessment**			
	Land Use	less than 2 km	2–5 km	5–10 km	Above 10 km
	Commercial Zone	24	20	56	6
**Access Mode**	Educational Zone	50	38.46	12	4
	Industrial Zone	30	32	28	1
	Commercial Zone	45	12	43	1
**Access sub- Mode**	Educational Zone	36	29	35	3
	Industrial Zone	43	26	31	1
	Commercial Zone	30	36	34	12
**Recreation**	Educational Zone	31	30.7	38	9
	Industrial Zone	29	30	41	7
	Commercial Zone	41	30	29	
**Circulation/Exchange**	Educational Zone	36	32	32	
	Industrial Zone	46	40	14

### 1.3 Bivariate analysis

The above table showed a positive correlation. This infers that there is a correlation between the age of the respondent to how often the responder walks (Table 3). This followed a similar trend with correlation of frequency of trip and income (Table 4).

**Table 3:** Bivariate analysis of frequency of walk trip and age of respondents.

		Walk Trip	Age
Frequency of walk trip	Pearson Correlation	1	.808^**^
	Sig. (2-tailed)		0
	N	472	472
Age	Pearson Correlation	.208^**^	1
	Sig. (2-tailed)	0	
	N	472	472

**Table 4:** Bivariate analysis of frequency of walk trip of respondents and income.

		Gender	level_of_education
Frequency of Walk Trip	Pearson Correlation	1	.584^**^
	Sig. (2-tailed)		0
	N	472	472
Income	Pearson Correlation	.584^**^	1
	Sig. (2-tailed)	0	
	N	472	472

## 2 Experimental design, materials and method

Ota a semi urban industrial area was used for data collection. This is the second most industrialized zone in South Western Nigeria. To achieve the aim of this research questionnaires were used for data collection using 1:15 sampling unit. The questionnaires were distributed evenly to five hundred respondents paying strict adhesion to ethics and confidentiality. The research focused on respondents aged 18 and above as they constitute a large percentage of the total population of the nation. The collected data was analyzed using descriptive method and bivariate analysis. This was also backed up with information from the focus discussion group. SPSS version 23 was used in the data analysis. It is important to note that need to provide transportation facilities for active transportation (walking and cycling) is necessary. Research of [1], [2], [3], [4], [5], [6], [7], [8], [9], [10], [11], [12], [13] assessed travel behaviors, such as trip-making frequency and distance and time traveled, have been studied for a variety of neighborhood types.
