# Supplementary material for: Non motorized trip pattern of high density neigbourhood: Data on demography and socio-economic parameters
Source: Data Brief. 2018 Aug 30;21:2658–63. doi: 10.1016/j.dib.2018.08.082 (PMC6290132; doi:10.1016/j.dib.2018.08.082)
Supplement: Supplementary file 1 — Supplementary material. [file mmc1.docx]

**Conflict of Interest**

*The authors have declared no conflict of interest*

Thanks
Yours faithfully,


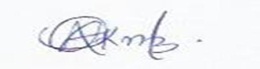


Busari, Ayobami.

**Corresponding author**
